# Getting knowledge to provide care: prevalence and factors associated with Sexually Transmitted Infections in immigrants from Goiás

**DOI:** 10.1590/1980-220X-REEUSP-2023-0034en

**Published:** 2024-01-08

**Authors:** Carla de Almeida Silva, Grazielle Rosa da Costa Silva, Thaynara Lorrane Silva Martins, Winny Éveny Alves Moura, Davi Oliveira Gomes, Gabriela Nolasco Bandeira, Megmar Aparecida dos Santos Carneiro, Roxana Isabel Cardozo Gonzalez, Leonora Rezende Pacheco, Margareth Santos Zanchetta, Juliana de Oliveira Roque e Lima, Sheila Araujo Teles, Karlla Antonieta Amorim Caetano

**Affiliations:** 1 Universidade Federal de Goiás, Faculdade de Enfermagem, Departamento de Enfermagem, Goiânia, GO, Brasil.; 2 Universidade Federal de Goiás, Instituto de Patologia Tropical e Saúde Pública, Departamento Virologia, Goiânia, GO, Brasil.; 3 Toronto Metropolitan University, Daphne Cockwell School of Nursing, Toronto, ON, Canadá.

**Keywords:** Sexually Transmitted Diseases, Emigration and Immigration, Refugees, Enfermedades de Transmisión Sexual, Emigración e Inmigración, Refugiados, Infecções Sexualmente Transmissíveis, Emigração e Imigração, Refugiados

## Abstract

**Objective:**

To estimate the prevalence of Sexually Transmitted Infections (STIs) in immigrants and refugees living in the metropolitan region of Goiânia, Goiás.

**Method:**

This is a cross-sectional and analytical study. Data collection was carried out from July 2019 to January 2020 and 308 immigrants and refugees were included in the sample. All were underwent face-to-face interviews and were tested for HIV, Syphilis, and Hepatitis B, using rapid tests.

**Results:**

The general prevalence for any of the STIs investigated was 8.8% (95%CI 6.0% – 12.3%), being 5.8% (95%CI 3.6% – 8.9%) for Hepatitis B, 2.3% for Syphilis (95%CI 1.00% – 4.4%) and 0.7% for HIV (95%CI 0.1% – 2.1%). Multiple analysis, using logistic regression, showed that the variables male gender (OR = 2.7) and length of time living in Brazil (OR = 2.6) were significantly associated with STIs (p < 0.05).

**Conclusion:**

The results of this study suggest that STIs are a health problem in immigrants/refugees, which appear to be enhanced with the length of migration in the country. Public policies that guarantee health care for this population shall be considered.

## RESUMO

**Objetivo:**

Estimar a prevalência de Infecções Sexualmente Transmissíveis (IST) em imigrantes e refugiados residentes na região metropolitana de Goiânia, Goiás.

**Método:**

Trata-se de um estudo transversal e analítico. A coleta de dados foi realizada no período de julho de 2019 a janeiro de 2020 e integraram a amostra 308 imigrantes e refugiados. Todos foram entrevistados face-a-face e testados para HIV, Sífilis e Hepatite B, por meio de testes rápidos.

**Resultados:**

A prevalência geral para alguma das IST investigadas foi de 8,8% (IC95% 6,0% – 12,3%), sendo 5,8% (IC95% 3,6% – 8,9%) para Hepatite B, 2,3% para Sífilis (IC95% 1,00% – 4,4%) e 0,7% para HIV (IC95% 0,1% – 2,1%). A análise múltipla, por regressão logística, mostrou que as variáveis sexo masculino (OR = 2,7) e tempo de moradia no Brasil (OR = 2,6) foram associadas significativamente às IST (p < 0,05).

**Conclusão:**

Os resultados deste estudo sugerem que as IST são um problema de saúde em imigrantes/refugiados, que parecem ser exacerbadas com o tempo de migração no país. Políticas públicas que garantam a assistência à saúde dessa população devem ser consideradas.

## RESUMEN

**Objetivo:**

Estimar la prevalencia de Enfermedades de Transmisión Sexual (ETS) en inmigrantes y refugiados residentes en la región metropolitana de Goiânia, Goiás.

**Método:**

Se trata de un estudio transversal y analítico. La recolección de datos se llevó a cabo desde julio de 2019 hasta enero de 2020 y se incluyeron en la muestra 308 inmigrantes y refugiados. Todos fueron entrevistados cara a cara y sometidos a pruebas de VIH, Sífilis y Hepatitis B, mediante pruebas rápidas..

**Resultados:**

La prevalencia general para cualquiera de las ETS investigadas fue de 8,8% (IC95% 6,0% – 12,3%), siendo 5,8% (IC95% 3,6% – 8,9%) para Hepatitis B, 2,3% para Sífilis (IC95% 1,00% – 4,4%) y 0,7% para VIH (IC95% 0,1% – 2,1%). El análisis múltiple, mediante regresión logística, mostró que las variables género masculino (OR = 2,7) y tiempo de residencia en Brasil (OR = 2,6) se asociaron significativamente con las ETS (p < 0,05).

**Conclusión:**

Los resultados de este estudio sugieren que las ETS son un problema de salud en inmigrantes/refugiados, que parecen exacerbarse con la duración de la migración en el país. Se deben considerar políticas públicas que garanticen la atención de la salud de esta población.

## INTRODUCTION

The 21st century has been marked by a high global migration flow and there are several causes that motivate this human circulation, such as wars and social, political, economic and environmental issues. A scenario of complexity, in search for better living conditions and survival in other nations^(1)^.

It is estimated that in 2021, around 281 million people were international immigrants^(2)^. According to data from the UN Refugee Agency (UNHCR), in mid-2022, the number of people forced to move from their homes reached 103 million, including refugees, asylum seekers, other people in need of international protection, and people internally displaced^(3)^. The UNHCR defines immigrants as people who move to improve their living, working and/or education conditions, while refugees are considered people who have fled persecution, armed conflicts, situations that are so dangerous that make them cross international borders in search for safety in other countries^(4)^.

In Brazil, the last decade (2011-2020) revealed a change in the immigrants profile and progress in receiving this population. In 2021, 151,155 immigrants were registered in Brazil^(5)^, as well as 29,107 requests for recognition of refugee status^(6)^. The number of people requesting asylum has increased significantly, with a growing process of feminization and an increase in the number of children and adolescents^(6)^.

Important and significant milestone for the immigrant and refugee population residing in Brazil was the approval of laws no. 9.474/1997 and no. 13.445/2017. The first one deals with the refugee status, with rights and duties to be granted to the stateless person or native of another country^(7)^. The second law guarantees rights to all immigrants and presents a condition of equality with national inhabitants, fighting discrimination^(8)^.

The difficulties faced by the foreign immigrant and refugee population go beyond migration procedures. When it comes to health, many variables can contribute to that population’s illness, including exposure to environmental, microbiological, and behavioral risk factors in the country of origin and low accessibility to health services in the country of destination, which hinders the obtainment of early diagnoses, or continuation of some specific treatment^(1)^. Infections caused by the human immunodeficiency virus (HIV), Hepatitis B virus (HBV), and Treponema pallidum (T. pallidum) have been the cause of high morbidity and mortality worldwide, and the greatest burden of these infections is observed on socially and economically vulnerable populations. These are sexually transmitted infections, but are also vertically spread. Furthermore, HIV and HBV are also efficiently transmitted parenterally^(9,10)^.

The epidemiology of these STIs, HIV, Syphilis, and Hepatitis B, reveals higher rates in low-income countries, where most immigrants and refugees generally come from. Furthermore, it should be highlighted that this population’s life in the destination country favors the continuity of the sexually transmitted infections^(11)^ transmission chain. The high consumption of alcohol, drugs, as well as risky sexual behaviors, such as not using condoms, are characteristics identified among the foreign immigrant and refugee population from various regions of the world^(12,13)^.

Brazil is a country with low endemicity for Hepatitis B, and the HIV epidemic is concentrated, that is, its highest frequency is observed in key populations^(14,15)^. Regarding Syphilis, the detection rate has increased in recent years, especially in adolescents and young adults^(16)^. To our knowledge, there are no studies on the diagnosis of STIs and their association with sexual health conditions among the foreign migrant population in Brazil. Knowing the epidemiological profile of HIV, Hepatitis B and Syphilis in an emerging population, such as immigrants and refugees, who have little access to public health services, contributes to the development of public healthcare policies that consider the specificities of different population groups. In addition, it contributes to international goals for eliminating AIDS and viral Hepatitis in the coming years and reduction in the incidence of syphilis^(17)^.

In the last decade, there has been an increase in the number of immigrants and refugees in Brazil in several Brazilian states, including the state of Goiás, coming mainly from Latin American countries such as Haiti, Bolivia, Colombia, Argentina, and Venezuela^(5,6)^. Therefore, this pioneering study aims to investigate the epidemiology of Sexually Transmitted Infections in immigrants and refugees living in the most populous region of Goiás: Goiânia and surrounding areas.

## METHOD

### Design of Study

Cross-sectional, analytical study.

### Local

Developed in the central region of the state of Goiás, in the cities of Goiânia, Aparecida de Goiânia, Senador Canedo and Anápolis.

### Population

The target population of the study consisted of immigrants/refugees assisted by religious leaders and civil society organizations.

### Selection Criteria

The inclusion criteria for this study were: having initiated sexual activity; presenting at least one of the requirements related to migratory status (immigrants in a vulnerable situation, refugees, immigrants for humanitarian reasons or environmentally displaced people, mixed migratory flows, stateless people); and being resident or traveling and/or passing through the central region of Goiás. An immigrant in vulnerable situations is a person who is a victim in situations that limit their ability to avoid and resist violence, exploitation, and abuse experienced during the migratory context; refugee is someone who left their country due to persecution or another situation affecting human rights; immigrants for humanitarian reasons are victims of human rights violations, such as human trafficking; environmental displacement,+ due to environmental disasters such as earthquakes; mixed migratory flows are population movements that include refugees, those displaced for environmental reasons, and economic immigrants; stateless persons are those who do not have a nationality^(4,18)^. Those immigrants or refugees residing in Goiás for more than 10 years were excluded.

### Sample Size Calculation

For sample calculation, in the absence of a regional parameter, an anti-HIV prevalence of 2.2% was considered, according to a population-based study carried out in Haiti, in individuals aged 15 to 49 years, period 2005-2006^(19)^, statistical power of 80% (β = 20%), significance level of 95% (α < 0.05), absolute precision of 3%, design effect 3, plus 10% for possible losses, requiring 303 participants. In the end, the sample consisted of 308 refugee immigrants.

### Data Collection

Data collection was carried out from July 2019 to January 2020. The team consisted of research assistants, researchers, and coordinators, linked to the Center for Studies in Epidemiology and Care in Transmissible Infections and Diseases (*NECAIH*) of the Nursing School (*FEN*) at the Universidade Federal de Goiás (*UFG*). For the interview stage, the project had the collaboration of translators with proficiency in French, English, Spanish, and Creole (Haiti).

Immigrants/refugees who agreed to participate in the project were informed about the importance of the research, the objectives, risks, benefits of participation, and their free will to interrupt participation at any time. After the clarifications, participants aged 18 years or over receveid the Free and Informed Consent Form (FICF) in Portuguese or in his/her language, in two copies, for reading and signing. For those under 18 years of age, the Free and Informed Assent Form (FIAF) was presented to both the participant and their legal representative and the Free and Informed Consent Form for children and adolescents participation.

All data collection instruments were developed in Portuguese, Spanish, and French Creole (Haiti). The interviews were carried out individually, in a private location, face-to-face, using a structured script containing questions regarding the sociodemographic and migratory characteristics, and risk behaviors for STIs. After this stage, samples for rapid tests (RT) were collected to detect anti-HIV, anti-*Treponema Pallidum,* and HBsAg.

For the anti-HIV test, two rapid tests were used. The first was the HIV 1/2/O Tri-line kit (Abon Biopharm (Hangzhou) Co Ltd. – China). This test detects HIV1 (gp41) and HIV2 (gp36) antigens, has sensitivity and specificity of 100% and 99.8%, respectively. The confirmatory test for HIV was the Dual Path Platform (DPP®) HIV 1/2 kit (Bio-Manguinhos). This test has a sensitivity of 100% and a specificity of 99.9%.

TR Alere™ Syphilis was used for anti-*T. Pallidum*. This test detects IgG, IgM, and IgA antibodies (qualitative) against the *T. pallidum*, in serum, plasma or whole blood, and has a sensitivity of 99.3% and specificity of 99.5%.

The identification of the infection marker of the Hepatitis B was through TR Bioclin-HBsAg. The method uses anti-HBsAg antibodies, which react with antigens present in serum, plasma and whole blood samples, and has a sensitivity of 99.9% and specificity of 99.8%.

The outcome variable considered was the presence of STI, determined by RT positivity for HIV and/or RT positivity for Syphilis, and/or RT positivity for Hepatitis B.

Independent variables: Sociodemographic situation (age, sex, marital status, years of life in Brazil, continent of origin, migration status in Brazil, education, desire to return to their country, receiving help in Brazil, religion, difficulties faced in Brazil) and Sexual behavior associated with exposure to STIs (alcohol use, sex professional, sexual intercourse under the influence of alcohol or other drugs, condom use in the last 12 months, initiation of sexual activity, drug use in the last 12 months, number of partners in the last 12 months, forced sexual intercourse, report of STI, sexual intercourse with a partner diagnosed with an STI, sexual intercourse with a partner who uses illicit drugs).

### Data Analysis and Treatment

Data were entered into Epidata SPSS software and analyzed using the SPSS statistical package version 24. Absolute and relative frequencies, measures of central tendency and prevalence with 95% confidence interval (95% CI) were calculated. The chi-square and Fisher’s exact tests were used to analyze differences between proportions, and the t-student and Mann-Whitney tests were used to analyze differences between means and medians. To identify the behaviors and characteristics associated with the outcome (presence of STI), the logistic regression model was used. Initially, bivariate analysis was performed and those variables that had p values <0.250 were used in the multiple analysis by logistic regression, using the Forward method as selection criteria. At the end, p values < 0.05 were considered significant.The model adjustment quality was carried out using the *Hosmer-Lemershow test* (p = 0.1199).

### Ethical Aspects

This study was approved by the Research Ethics Committee of the Universidade Federal de Goiás with opinion number 3.243.845, approved in 2019, and by the Research Ethics Board of the Toronto Metropolitan University, with no. 166-2019.27. The study is in compliance with Resolution 466/2012 and the General Data Protection Law – LGPD – no. 13.709, of August 14, 2018.

## RESULTS

The general prevalence of STIs studied among immigrants and refugees was 8.8% (n = 27/308; 95%CI 6.0% – 12.3%). The prevalence of Hepatitis B was 5.8% (n = 18/308; 95% CI 3.6% – 8.9%), Syphilis 2.3% (n = 7/308; 95% CI 1.00% – 4.4%), and HIV of 0.7% (n = 2/308; 95% CI 0.1% – 2.1%). No co-infection was observed, considering the pathogens investigated.

### Bivariate Analysis of Sociodemographic and Immigration Factors Associated with Exposure to STIs

Table 1 presents the bivariate analysis of sociodemographic and immigration factors associated with exposure to STIs in the study sample. The following variables presented p < 0.05 and were associated with the presence of STIs among foreign immigrants and refugees: sex (p = 0.025), years in Brazil (p = 0.024), and age (p = 0.027). Regarding country of birth, 209 (67.8%) were immigrants and refugees from Haiti, 81 (26.3%) were from Venezuela, 14 (5.8%) from Guinea Bissau, and 0.1% were from Colombia, Cuba, Ecuador, and Spain.

**Table 1:** Bivariate analysis of sociodemographic and immigration factors associated with exposure to STIs in 308 immigrants in Goiânia and its Metropolitan Region – Goiânia, GO, Brazil, 2019–2020.

Variables	Bivariate analysis			
	Exposure to STIs (n = 308)			
	Total	Positive	Negative	*p-value*
	n = 308(%)	n = 27(%)	n = 281 (%)	
**Sex**				*0.025*
Male	175 (56.8)	21 (12.0)	154 (88)	
Female	133 (43.2)	6 (4.5)	127 (95.5)	
Marital status (NI:1)***				*0.687*
Single/Separated/Widowed	140 (45.8)	11 (7.9)	129 (92.1)	
Married/Living together	166 (54.2)	16 (9.6)	150 (90.4)	
**Years in Brazil**				*0.024*
≤1	171 (55.5)	9 (5.3)	162 (94.7)	
≥2	137 (44.5)	18 (13.1)	119 (86.9)	
Age* 33.8(9.5)**				*0.027*
16-33 years old	166 (53.9)	9 (5.4)	157 (94.6)	
34-64 years old	142 (46.1)	18 (12.7)	124 (87.3)	
**Continent**				*0.665*
South America	102 (33.1)	7(6,9)	95 (93.1)	
Central America	192 (62.3)	19 (9.9)	173 (90.1)	
Africa	14 (4.5)	1 (7.1)	13 (92.9)	
**City of residence**				*0.704*
Anápolis	28 (9.1)	2 (7.1)	26 (92.9)	
Aparecida de Goiânia	133 (43.2)	10 (7.5)	123 (92.5)	
Goiânia	42 (46.1)	15 (10.6)	127 (89.4)	
Senator Canedo	5 (1.6)	0 (0)	5 (100.0)	
**Situation in Brazil**				*0.532*
Immigrant	236 (76.6)	22 (9.3)	214 (90.7)	
Refugee	72 (23.4)	5 (6.9)	67 (93.1)	
**Continent**				*0.665*
South America	102 (33.1)	7(6,9)	95 (93.1)	
**Professional situation in Brazil**				*0.076*
Formal or informal employee	129 (41.9)	16 (12.4)	113 (87.6)	
Unemployed	63 (20.5)	4 (6.3)	59 (93.7)	
Student	116 (37.7)	7 (6.0)	109 (94.0)	
Education* 11.2(5.2)** (NI: 23)***				
≤9 years	71 (24.9)	9 (12.7)	62 (87.3)	
10-12 years	90 (31.6)	4 (4.4)	86 (95.6)	
≥13 years	124 (43.5)	13 (10.5)	111 (89.5)	
Wants to return to his/her country (NI:1)***				*0.876*
No	104 (33.9)	10 (9.6)	94 (90.4)	
Yes	160 (52.1)	14 (8.8)	146 (91.3)	
Do not know	43 (14.0)	3 (7)	40 (93)	
Receiving aid in Brazil (NI:1)***				*0.998*
No	182 (59.3)	16 (8.8)	166 (91.2)	
Yes	125 (40.7)	11 (8.8)	114 (91.2)	
**Religion**				*0.664*
No	18 (5.8)	2 (11.1)	16 (88.9)	
Yes	290 (94.2)	25 (8.6)	265 (91.4)	
Difficulties faced in Brazil (NI:1)***				
No difficulty	44 (14.3)	2 (4.5)	42 (95.5)	*0.153*
With the Portuguese language	239 (77.9)	22 (9.2)	217 (90.8)	
In finding a job	2 (0.7)	1 (50.0)	1 (50.0)	
Others (Climate, food, prejudice, discrimination)	22 (7.2)	2 (9.1)	20 (90.9)	

*Years.

**Mean (standard deviation).

*** NI: No information.

### Bivariate Analysis of Sexual Behavior Factors Associated with Exposure to STIs

Table 2 presents the bivariate analysis of sexual behavior factors associated with exposure to STIs in the study sample. No variable was associated with the outcome.

**Table 2:** Bivariate analysis of sexual behavior factors associated with exposure to STIs in 308 immigrants in Goiânia and its Metropolitan Region – Goiânia, GO, Brazil, 2019–2020.

Variables	Bivariate analysis			
	Exposure to STIs (n = 308)			
	Total	Positive	Negative	*p-value*
	n = 308(%)	n = 27(%)	n = 281 (%)	
Alcohol use (NI:4)***				*0.542*
No	134 (44.1)	13 (9.7)	121 (90.3)	
Yes	170 (55.9)	13 (7.6)	157 (92.4)	
Sex worker (NI:2)***				*0.570*
No	297 (97.1)	26 (8.8)	271 (91.2)	
Yes	9 (2.9)	1 (11.1)	8 (88.9)	
Sexual intercourse under the influence of alcohol or other drugs (NI:2)***				*1*
No	271 (88.6)	24 (8.9)	247 (91.1)	
Sim	35 (11.4)	3 (8.6)	32 (91.4)	
Condom use in the last 12 months (NI:50)***				*0.794*
Yes	53 (20.5)	4 (7.5)	49 (92.5)	
No	206 (79.5)	21 (10.2)	185 (89.8)	
Sexual activity initiation* 16.7(4.0)** (NI:32)***				*0.536*
≤16 years old	128 (46.4)	10 (7.8)	118 (92.2)	
≥17 years	148 (53.6)	15 (10.1)	133 (89.9)	
Drug use in the last 12 months (NI:1)***				*0.242*
No	304 (99.0)	26 (8.6)	278 (91.4)	
Yes	3 (1.0)	1 (33.3)	2 (66.7)	
Number of partners in the last 12 months (NI:12)***				*0.590*
≤1	244 (82.4)	23 (9.4)	221 (90.6)	
≥2	52 (17.6)	3 (5.8)	49 (94.2)	
Forced sexual intercourse (NI:2)***				*0.152*
No	278 (90.8)	27 (9.7)	251 (90.3)	
Yes	28 (9.2)	0 (0.0)	28 (100.0)	
Report of STI (NI:1)***				*0.135*
No	292 (95.1)	24 (8.2)	268 (91.8)	
Yes	15 (4.9)	3 (20.0)	12 (80.0)	
Sexual intercourse with a partner diagnosed with STI (NI:1)***				*1*
No/ Doesn’t know	299 (97.4)	27 (9.0)	272 (91.0)	
Yes	8 (2.6)	0 (0)	8 (100.0)	
Sexual relationship with a partner who uses illicit drugs (NI:5)***				*1*
No/ Doesn’t know	301 (99.3)	27 (9.0)	274 (91.0)	
Yes	2 (0.7)	0 (0)	2 (100.0)	

*Years.

**Mean (standard deviation).

***NI: No information.

### Multiple Analysis of Variables Associated with Exposure to STIs

After logistic regression analysis, the following variables remained significantly associated with the outcome and represented risk for exposure to STIs (p < 0.05): sex (OR = 2.7) and years in Brazil (OR= 2.6) (Table 3).

**Table 3:** Multiple analysis of sociodemographic and immigration variables associated with exposure to STIs in 308 immigrants in Goiânia and its Metropolitan Region – Goiânia, GO, Brazil, 2019–2020.

Variables	Multiple analysis			
	Exposure to STIs (n = 308)			
	Positive	Negative	*p-value*	OR* (95% CI)
	n = 27 (%)	n = 119 (%)		
**Sex**				
Female	6 (4.5)	127 (95.5)		
Male	21 (12.0)	154 (88)	*0.037*	*2.7 (1.1–7.0)*
**Years in Brazil**				
≤1	9 (5.3)	162 (94.7)		
≥2	18 (13.1)	119 (86.9)	*0.027*	*2.6 (1.1–6.0)*

*OR: Odds ratio; 95% CI: 95% confidence interval.

## DISCUSSION

In Brazil, this is the first seroepidemiological study that identified the prevalence of STIs in immigrants and refugees. The rate found is higher than the prevalences identified in the general population of the country^(14-20)^ and similar to that of socially vulnerable groups living in different regions of Brazil^(21,22)^.

At an international level, what is observed is a varied prevalence of sexually transmitted infections among immigrants and refugees around the world. In our study, there was a predominance of Haitians and Venezuelans. In these countries of origin, high rates of HIV, Hepatitis B, and Syphilis are identified among the population^(23,24)^. Thus, the migratory influx can impact the detection rate of infections in the country that received them, either through the introduction of cases when entering the country, or through adverse living conditions that favor the acquisition and dissemination of infectious diseases in an emerging population that lives on the sidelines of the health service^(25)^.

From this perspective, knowing the risk factors for STIs in refugee immigrants can contribute to breaking the chain of transmission of sexual diseases and consequently support actions to control and prevent these infections in our country.

In the present study, being male was independently associated with all the STIs investigated. Other studies have shown the association between sex and STIs. In the context of migration, in many cases, men are the first in their families to move and the migratory process can favor unprotected sexual practices and multiple partnerships that constitute a risk for STIs^(11)^.

Our data also suggest that the time of migration in Brazil contributed to the acquisition of STIs. Although our study is cross-sectional, which prevents us from inferring causality, it should be noted that the age cohort effect was controlled in our multiple regression model. Thus, we can speculate that immigrants and refugees, due to risky sexual habits and behaviors maintained or acquired after arrival in Brazil, are exposing themselves to sexually transmitted infections^(11)^.

In addition to the vulnerable situations experienced in the migration process that corroborate the acquisition of these infections^(12)^, low adherence to condoms during sexual intercourse was observed. Of the total number of participants, only 17.2% reported sex with a condom, and 16.2% did not want to inform, which may be a reason for cultural questions among the participants. This problem suggests further investigation, as it is not possible to state that there is acculturation, a time in which many adapt and modify their reality in the new country, and that such change alters personal behaviors that predispose them to sexual risks^(11)^, or whether low adherence to condoms is an inherent habit of the migrant population. The fact is that this behavior requires intervention and knowing the reasons that lead to non-adherence is the initial step towards effective health education.

On the other hand, structural failures of assistance and low access to health services are the main challenges in reaching the immigrant/refugee population in Brazil. Certainly, the language barrier is one of the main limitations to access and use of health services^(26)^. Several strategies have been discussed for preventing and controlling STIs among immigrants and refugees. The people’s health status screening upon arrival in host countries is the cornerstone in controlling the entry of communicable diseases, and must include sexually transmitted diseases^(11)^.

In Brazil, operation *Acolhida* (Embracement), since 2019, has been working with various emergency assistance actions for Venezuelans crossing Rondônia’s border, such as provision of documentation, vaccination, and screening^(27)^. Conversely, this strategy cannot be seen as unique, because as pointed out in our study, experience in the destination country can be a risk factor. To face the various challenges, the global pact for immigration is addressed by the 2030 Agenda for Sustainable Development. Several commitments were signed by the Member States of the United Nations that, in short, assume the duty of international cooperation and its legal ethical aspects. Countries around the world are in this fight, building and executing actions according to local needs and reality^(28)^.

This study has some limitations. The convenience sampling used may compromise external validity. On the other hand, official data confirm, in recent years, the predominance of migration of Haitians and Venezuelans in our country^(5,6)^, confirming the representativeness of the sample. Furthermore, this is a sample made up predominantly of practicing religious people, with strong moral values, which may have contributed to the existence of response biases, since the questions addressed sexual behaviors. However, the interviewers were trained to provide privacy and safety to the participant for greater veracity of the information received. This study contributes to the advancement of care for this vulnerable population, improving knowledge about their health profile, enhancing quality of life and access to health services guaranteed by law.

## CONCLUSION

The study shows a high prevalence of STIs in immigrants/refugees, compared to the Brazilian general population, and suggests that STIs are a health problem in this population and seem to be exacerbated with the time of migration in Brazil. Public policies that guarantee health care for this population must be considered, such as reception programs and health services capable of meeting the specificities of immigrants/refugees living in our country.
